# Transplacental Toxicity of Zinc Oxide Nanoparticles: Maternal-Fetal DNA Damage and Organ Accumulation

**DOI:** 10.3390/nano16150967

**Published:** 2026-08-06

**Authors:** Elsayed I. Salim, Naira M. Al-Fiky, Khaled Y. Abdel-Halim, Dina M. M. AlSadek, Haitham A. Badr, Mohamed Monir Hammad, Hassan A. Basha, Fouad A. Abou-Zaid, Hafiz Ahmed

**Affiliations:** 1 Research Laboratory of Molecular Carcinogenesis, Department of Zoology, Faculty of Science, Tanta University, Tanta 31527, Egypt; elsayed.salim@science.tanta.edu.eg (E.I.S.); nora.fiky@gmail.com (N.M.A.-F.); hassan.basha878@gmail.com (H.A.B.); fouad.refae@science.tanta.edu.eg (F.A.A.-Z.); 2 Department of Mammalian & Aquatic Toxicology, Central Agricultural Pesticides Laboratory (CAPL), Agricultural Research Center (ARC), Dokki, Giza 12618, Egypt; khaled_yassen68@yahoo.com; 3 Department of Histology and Cytology, Faculty of Veterinary Medicine, Zagazig University, Zagazig 44511, Egypt; dina.alsadek@gmail.com; 4 Department of Biochemistry, Faculty of Agriculture, Zagazig University, Zagazig 44511, Egypt; egy.hab@gmail.com; 5 Department of Zoology, Faculty of Science, Alexandria University, Alexandria 21568, Egypt; m.mouner92@gmail.com; 6 GlycoMantra Inc., 1450 South Rolling Road, Baltimore, MD 21227, USA

**Keywords:** zinc oxide nanoparticles, nanotoxicology, transplacental transfer, maternal–fetal toxicity, oxidative DNA damage, 8-hydroxydeoxyguanosine (8-OHdG), organ accumulation

## Abstract

Nanomaterials (NMs) offer substantial technological advantages, yet their potential adverse biological effects remain a critical concern. This study investigates the maternal–fetal toxicity of zinc oxide nanoparticles (ZnONPs) following a single intravenous (i.v.) administration in vivo, focusing on oxidative DNA damage, cytotoxicity, and transplacental transfer. The median lethal dose (LD_50_) of ZnONPs was determined to be 154 mg/kg of body weight. Pregnant rats on gestational day 19 were exposed to two sub-lethal doses (3.09 and 7.71 mg/kg; corresponding to 1/50 and 1/20 of LD50). Subsequent analyses assessed Zn^2+^ accumulation, histopathological alterations in maternal organs, and induction of 8-hydroxydeoxyguanosine (8-OHdG) in maternal and fetal tissues. ZnONPs demonstrated systemic distribution, with pronounced accumulation in the liver, spleen, and placenta, and were shown to cross the placental barrier, leading to fetal exposure. Elevated Zn^2+^ concentrations were positively correlated with cytotoxicity and 8-OHdG induction across maternal and fetal compartments. These findings provide compelling evidence of ZnONPs-mediated cyto- and genotoxicity in both mothers and offspring, underscoring the need to define safety margins and regulatory thresholds for nanomaterial exposure in biomedical and environmental contexts.

## 1. Introduction

Metallic nanoparticles (MNPs), synthesized from pure metal precursors, have emerged as versatile materials with unique physicochemical and optical properties that underpin their widespread application across diverse scientific and industrial domains [1]. Structurally, MNPs typically consist of metallic or metal oxide cores, often encapsulated within oxide shells or stabilized by organic and inorganic coatings. Their integration into consumer products, including textiles, packaging, cosmetics, and personal care items, has expanded rapidly, alongside their use in pharmaceuticals, food additives, pigments, semiconductors, sensors, and therapeutic agents [2,3].

Despite these technological advances, growing evidence highlights the potential adverse biological and environmental consequences of nanomaterials (NMs). Manipulation of matter at the nanoscale has been linked to toxicological outcomes, including oxidative stress, apoptosis, and inflammation, which collectively raise concerns regarding their biosafety [4]. Embryo–fetal developmental toxicity induced by nanoparticles (NPs) is influenced not only by maternal physiological conditions but also by the physicochemical characteristics of NPs and exposure parameters such as dose and timing [5].

Zinc oxide nanoparticles (ZnONPs) have been reported to exert detrimental effects across biological systems ranging from plants and microorganisms to vertebrates [6]. ZnONPs can enter organisms via multiple routes—including inhalation, dermal absorption, ingestion, and parenteral administration—and their ubiquity in the environment further increases human exposure risk [7]. In vitro studies have demonstrated ZnONP-induced oxidative stress, mitochondrial dysfunction, and disruption of protein and microRNA expression critical for neural development in astrocytes and neuronal cells [8]. Complementary in vivo investigations confirm neurotoxic outcomes, supporting the hypothesis that ZnONPs accumulate in distinct tissues depending on route and dose of administration [9].

Of particular concern is the ability of nanoparticles to traverse the placental barrier, thereby directly impacting fetal development [10]. Previous studies have associated prenatal ZnONPs exposure with reduced fetal weight, intrauterine growth retardation, altered blood pressure regulation, and neurodevelopmental deficits, including impaired learning, memory dysfunction, motor coordination deficits, and depressive-like behaviors [11]. These findings underscore the urgent need to delineate the mechanisms underlying ZnONPs-mediated maternal–fetal toxicity.

Accordingly, the present study investigates the potential induction of DNA damage and cytotoxicity by ZnONPs following a single intravenous (i.v.) administration in pregnant rats. Specifically, we examine the extent of transplacental transfer, organ-specific distribution, and the correlation between Zn^2+^ accumulation, histopathological alterations, and oxidative DNA damage (8-hydroxydeoxyguanosine, 8-OHdG) in both maternal and fetal tissues. This work aims to provide mechanistic insights into ZnONP-mediated genotoxicity and cytotoxicity, thereby contributing to the establishment of safety margins and regulatory frameworks for nanomaterial exposure during pregnancy.

## 2. Material and Methods

### 2.1. Chemicals

Zinc oxide nanoparticles (ZnONPs) were synthesized in the Nanotech Laboratory for Photoelectronics (City of 6 October, Giza, Egypt) as previously described [12]. Briefly, Zinc acetate dihydrate was reacted with potassium hydroxide in an alcoholic medium under low-temperature conditions, leading to the formation of ZnO nanoparticles via alkaline precipitation. A 1% citrate buffer was prepared by dissolving citric acid in ultrapure Milli-Q^®^ deionized water, then sonicating until a clear solution was obtained. Phosphate-buffered saline (PBS; pH 7.2) was prepared at 0.01 M concentration by dissolving salts in 1 L of deionized water at 25 °C. Specifically, NaCl (1.613 g; 0.138 M), KCl (0.0403 g; 0.0027 M), KH_2_PO_4_ (0.1369 g; 0.01 M), and K_2_HPO_4_ (0.174 g; 0.01 M) were dissolved separately in 200 mL of deionized water and combined to yield the final buffer solution. Nitric acid (HNO_3_; 69%) and hydrochloric acid (HCl; 30–34%) were obtained from S.D. Fine-Chem Limited (Mumbai, India). Hydrogen peroxide (H_2_O_2_; 30%) was purchased from Central Drug House (New Delhi, India). All experiments were conducted using Milli-Q^®^ ultrapure deionized water to ensure reagent purity and reproducibility.

### 2.2. Characterization of ZnONPs

The prepared ZnONP suspension was analyzed using a SPECTRO UV-VIS Dual Beam 8 Auto Cell spectrophotometer (LABOMED, Inc., Fremont, CA, USA) over the wavelength range of 190–1100 nm to determine the maximum absorption. Elemental composition was assessed by energy-dispersive X-ray spectroscopy (EDX) [13].

Samples were exposed to X-ray beams, and signals were detected using an X-Max 80 detector (Oxford Instruments, Abingdon, UK) coupled with a JEM-1230 scanning electron microscope (SEM) (JEOL Ltd., Tokyo, Japan). Crystallographic structure was examined by X-ray diffraction (XRD) using an APD 2000Pro Powder Diffractometer (APD 2000 Pro by GNR, Agrate Conturbia, Italy). Zeta potential measurements (−200 to +200 mV) were obtained with a Zetasizer Nano ZS (Malvern Instruments Ltd., Malvern, Worcestershire, UK), with three independent recordings per sample.

Chemical bonding and molecular structure were characterized by Fourier-transform infrared spectroscopy (FTIR) using a Bruker TENSOR spectrophotometer (Bruker Corporation, Billerica, MA, USA). For transmission electron microscopy (TEM), one drop of dispersed ZnONP suspension was placed on a carbon-coated copper grid. Morphology and particle size were evaluated using a JEOL JEM-100 SX TEM (JEOL Ltd., Tokyo, Japan) operated at an accelerating voltage of 120 kV.

### 2.3. Experimental Design

#### 2.3.1. Animal Husbandry

Eight-week-old female Sprague–Dawley rats were obtained from the Holding Company for Biological Products and Vaccines (Vaccera, Egypt). Animals were housed under standard laboratory conditions with a 12 h light/dark cycle and ad libitum access to food and water. A one-week acclimatization period was provided prior to experimentation. All procedures were approved by the Institutional Animal Care and Use Committee (IACUC-SCI-TU-0431).

#### 2.3.2. Intravenous Toxicity Assessment

Intravenous (i.v.) administration was employed to evaluate ZnONP toxicity in female rats (140 ± 20 g). The administered doses (3.09 and 7.71 mg/kg bw) were selected based on a preliminary pilot study to determine the LD_50_ in accordance with OECD Guideline 401 [14] and represent sublethal fractions (1/50 and 1/20 of LD_50_), while gestational day 19 was chosen as a late stage of pregnancy with a fully developed placenta and differentiated fetal organs.

Rats were divided into four groups and administered ZnONPs at 50, 100, 200, and 400 mg/kg of body weight, calculated using a factor of 2.0. The control group received a citrate buffer vehicle. Mortality was recorded daily, and deceased animals were removed. Clinical signs of toxicity were documented throughout the study. LD_50_ values were estimated using Finney’s method [15].

#### 2.3.3. Maternal Exposure and Sample Collection

For mating, female rats were housed two per cage with one male. Vaginal plugs were checked after 24 h, and the presence of a plug was considered day 0 of pregnancy. Females were then separated, and maternal body weights were recorded on gestation days 0 and 6.

Pregnant rats received ZnONPs at two sublethal doses (3.09 and 7.75 mg/kg bw; 1/50 and 1/20 of LD_50_, respectively) via a single intravenous injection into the lateral tail vein on gestation day 19. Animals were sacrificed at 1, 6, 12, and 24 h post-injection.

Maternal blood was collected, allowed to clot at room temperature, and centrifuged at 3000 rpm for 15 min to obtain serum. Amniotic fluids were also collected. Organs, including the liver, kidneys, spleen, placenta, uterus, and embryos, were harvested for subsequent analyses.

#### 2.3.4. Determination of Zn^2+^ Accumulation in Organs and Body Fluids

##### Sample Preparation

Serum, amniotic fluid, liver, kidney, spleen, uterus, placenta, and fetal tissues were collected. Organs were sliced, dried at 50 °C for 12 h, and ashed (0.1–0.5 g). Samples were digested with 10 mL of nitric acid under sonication until clear, then 10 drops of H_2_O_2_ were added. After cooling, 5 mL of deionized water was added, and the solution was filtered through a 0.45 µm filter. Final extracts were diluted to a final volume of 40 mL with deionized water [16].

##### Metal Ion Measurement

Zinc concentrations were quantified using inductively coupled plasma optical emission spectroscopy (ICP-OES; Optima 7000, Perkin Elmer, Shelton, CT, USA). An autosampler introduced samples into a cyclonic spray chamber with a mass-flow-controlled nebulizer gas flow of 0.5 L/min. The instrument operated in fast sequential mode with a per-liter cooled CCD detector, and background/spectral interferences were corrected using Expert Software (version 3.0., HORIBA Ltd., Kyoto, Japan).

Limits of detection (LOD) and quantification (LOQ) were calculated twice the standard deviation of blank measurements. Working standards and recovery experiments (spiked blanks at two levels) ensured accuracy. All samples were handled under strict quality assurance protocols, using ultrapure deionized water to prevent contamination.

#### 2.3.5. Histopathological Analysis (H&E Staining)

Liver, kidney, spleen, and placental tissues were excised and fixed in 10% phosphate-buffered formalin for at least 72 h. Following fixation, specimens were rinsed with tap water, dehydrated through graded alcohols, cleared in xylene, and embedded in paraffin wax at 55 °C. Serial sections (6 μm) were prepared and stained with hematoxylin and eosin for light microscopic examination.

#### 2.3.6. Transmission Electron Microscopy (TEM)

Spleen and placental specimens collected at 12 and 24 h post-treatment were fixed in 4% glutaraldehyde in 0.1 M sodium cacodylate buffer (pH 7.3) for 6 h, followed by overnight buffer washes. Post-fixation was performed in 1% osmium tetroxide for 2 h. Semi-thin sections (1 μm) were stained with toluidine blue for preliminary examination. Ultra-thin sections (70–100 nm) were cut using an LKB ultramicrotome, mounted on copper grids, and stained with uranyl acetate (30 min) and lead citrate (15 min). Sections were examined with a JEOL 100S TEM at 80 kV.

### 2.4. Quantification of 8-OHdG by ELISA

Maternal blood, liver, spleen, placenta, and fetal samples collected at 1, 6, 12, and 24 h were processed by centrifugation (2800–4500× *g*, 20 min). Tissue homogenates were prepared in PBS (pH 7.4) and centrifuged similarly. Supernatants were analyzed using rat 8-OHdG ELISA kit (MyBioSource, Inc., San Diego, CA, USA. Catalog #: MBS267513). Aliquots (40 μL) were added to antibody-coated microtiter plates, followed by 10 μL monoclonal antibody and 50 μL streptavidin-HRP. Plates were incubated at 37 °C for 60 min, washed 5 times, and sequentially treated with chromogen solutions A and B. After 10 min incubation, reactions were stopped with 50 μL stop solution, and absorbance was read at 450 nm (Bio-Rad PR 4100, Hercules, CA, USA). Triplicate measurements were performed, with CV < 10%. Standard curves were generated, and 8-OHdG levels were expressed as ng/mL or ng/g tissue. LOQ ranged from 0.05 to 20 ng/mL; LOD was 0.027 ng/mL.

### 2.5. Statistical Analysis

LD_50_ values (mg/kg body weight) with confidence limits and slope were calculated using probit analysis [15]. Data (mean ± SE) were analyzed by ANOVA, with significance determined by LSD at *p* ≤ 0.05 (CoStat Version 3, Cohort Software, Tucson, AZ, USA). Linear regression was performed, with * *p* ≤ 0.05 and ** *p* ≤ 0.01 significance levels (Fisher distribution; StatistXL v2.1). Histopathological differences between groups were further evaluated using box-and-whisker plots (SPSS v16).

## 3. Results

### 3.1. Physicochemical Characterization of ZnONPs

The UV–Vis’s absorbance spectrum of the sample was recorded over the wavelength range 190–1000 nm. A pronounced absorption peak is observed at λmax 258 nm, attributed to the characteristic electronic transition of zinc ions. The spectrum remains relatively flat in the visible and near-infrared regions, indicating high optical transparency (Figure 1a). EDX analysis verified nanoparticle formation and elemental composition, showing ~73.5% Zn, 22.5% O, 2.5% Al, and 1.4% Cu, indicating high purity (Figure 1b). XRD analysis identified diffraction peaks corresponding to ZnO hexagonal wurtzite crystal planes (ICDD card no. 01-089-1397), with peak broadening confirming nanoscale crystallinity (Figure 1c).

Zeta potential analysis showed an average value of −8.05 mV (Figure 1d). FTIR spectra displayed a Zn–O absorption band at 533 cm^−1^ and a broad hydroxyl peak at 3398 cm^−1^ (Figure 1e). TEM imaging revealed predominantly spherical ZnONPs with diameters ranging from 21.6 to 46.1 nm (mean 36.8 nm) (Figure 1f).

### 3.2. Acute Toxicity and LD_50_ Determination

No overt toxicological signs or gross pathological changes were observed during the study. The LD_50_ of ZnONPs was calculated as 154 mg/kg bw, with slope 1.9503, variance 0.2122, and confidence limits of 131.7–181.7 mg/kg bw.

### 3.3. Zn^2+^ Accumulation in Maternal and Fetal Compartments

At an administration (i.v.) of 3.09 mg/kg bw, Zn^2+^ was widely distributed across maternal and fetal tissues (Figure 2; see also Table 1 for the Zn^2+^ levels across most organs at the time points). At 1 h, spleen (50.7 µg/g), kidneys (49.4 µg/g), uterus (46.5 µg/g), and embryos (40.7 µg/g) showed moderate accumulation. By 6 h, Zn^2+^ content decreased significantly, except in the uterus (65.2 µg/g). Liver Zn^2+^ peaked again at 12 h (40.7 µg/g) before dropping to 15.2 µg/g at 24 h. Amniotic fluid showed consistently low levels, except at 24 h (43.6 µg/mL; *p* < 0.05).

At 7.71 mg/kg bw (Table 2), the liver exhibited the highest Zn^2+^ accumulation (44.3 µg/g at 12 h; 38.3 µg/g at 24 h). Kidney’s concentrations declined progressively (30.0 → 24.1 → 17.4 → 19.5 µg/g across 1–24 h). Serum showed the lowest accumulation (19.2 → 9.9 → 7.4 → 4.0 µg/mL). Placenta displayed early high levels (20.3 µg/g at 1 h), followed by a sharp decline to 0.1 µg/g at 12 h (Table 2).

### 3.4. Transplacental Transfer of Zn^2+^

At 3.09 mg/kg bw administration, the fetal Zn^2+^ concentrations peaked at 40.8 ± 0.8 µg/g (3.3% of dose) after 1 h, then declined to 3.0 ± 5.1 µg/g (0.30%) at 6 h. Levels stabilized at 17.4 µg/g (1.58%) and 13.5 µg/g (1.26%) at 12 and 24 h, respectively. At 7.71 mg/kg bw, fetal Zn^2+^ reached 23.2 µg/g (2.06%) at 1 h, decreased to 13.1 µg/g (1.24%) and 5.7 µg/g (0.57%) at 6 and 12 h, and rose again to 23.2 µg/g at 24 h (Table 3). Figure 3 illustrates the inverse relationship between maternal blood Zn^2+^ and fetal tissue accumulation, with early fetal uptake followed by decline and late resurgence.

### 3.5. Histopathological Findings

#### 3.5.1. Liver

Control livers displayed normal lobular architecture. ZnONP exposure induced disorganization, hepatocyte vacuolation, cytoplasmic pallor, and nuclear darkening (Figure 3a–c).

#### 3.5.2. Spleen

No alterations were observed up to 6 h post-treatment. At 12–24 h, macrophages contained black spherical inclusions consistent with ZnONPs, alongside golden-brown deposits resembling hemosiderosis or erythrophagocytosis (Figure 3d–f).

#### 3.5.3. Placenta

Control placentas were intact. At 24 h post-exposure, spongiotrophoblast nuclei showed minor DNA fragmentation, focal necrosis, and cytoplasmic vacuolization, absent in controls (Figure 3g–i).

### 3.6. Ultrastructural Analysis

#### 3.6.1. Placenta

The TEM investigation revealed putative ZnONP deposits in trophoblasts at 12 h, absent in controls (Figure 4a–c). The placenta from rats i.v. administered with 3.09 mg/kg of ZnONPs and sacrificed after 12 h showed necrotic areas containing ZnONP deposits of varying sizes in spongiotrophoblasts. Placental trophoblasts of pregnant rats administered with 7.71 mg/kg of ZnONPs and sacrificed after 12 h showed necrotic properties such as nuclear membrane indentation, chromatin condensation, and pyknotic nuclei.

#### 3.6.2. Spleen

The control spleens appeared intact. At 12–24 h, ZnONP-treated spleens from the rats i.v. administered with 3.09 mg/kg of ZnONPs and sacrificed after 12 h showed presumed localized nanoparticles, while spleen sections from the rats treated with 7.71 mg/kg of ZnONPs and sacrificed after 12 h showed localized nanoparticles and exhibited macrophage vacuolization, necrosis, mitochondrial cristae loss, and nuclear indentation (Figure 4d–f).

#### 3.6.3. Nanoparticle Localization and Quantitative Morphometry

ZnONPs were observed within macrophages and lysosomes, ranging from 10.9 to 21.8 nm (3.09 mg/kg) and from 21.8 to 65.3 nm (7.71 mg/kg) (Figure 4e,f). Quantitative morphometry revealed dose-dependent changes in lysosomal surface area, nuclear dimensions, mitochondrial size, and cytoplasmic volume in the placenta and spleen (Figure 5a–e).

The surface area of nanoparticles (NPs) engulfed within lysosomes measured 0.125 and 0.164 µm^2^ at doses of 3.09 and 7.71 mg/kg, respectively, in placental sections. In contrast, splenic sections showed values of 1.013 and 0.285 µm^2^ for the same doses (Figure 5a). Similarly, the integrated density of precipitated NPs reached 39.555 and 61.137 in placental sections, compared to 261.971 and 30.012 in splenic sections at the respective doses (Figure 5b). In control samples, nuclear surface area in the placenta was 0.905 µm^2^, compared with 0.396 and 0.717 µm^2^ at doses of 3.09 and 7.71 mg/kg, respectively. Conversely, in splenic control sections, the nuclear surface area was lower (0.339 µm^2^) than in treated samples, which showed values of 0.613 and 1.326 µm^2^ at the corresponding doses (Figure 5c).

Cytoplasm/nucleus (Cyto/N) ratios declined significantly in the spleen (control: 6.22; 3.09 mg/kg: 3.91; 7.71 mg/kg: 1.33), correlating with Zn^2+^ levels (R^2^ = 0.999). Placental Cyto/N ratios showed minimal change (control: 5.74; 3.09 mg/kg: 5.74; 7.71 mg/kg: 3.76; R^2^ = 0.743). Cytoplasm/mitochondria (Cyto/M) ratios also shifted, with spleen showing significant correlation to ZnONP levels (R^2^ = 0.839 *), while placenta did not (R^2^ = 0.005) (Figure 6).

### 3.7. 8-Oxo-2’-Deoxyguanosine (8-OHdG) Induction

Quantified 8-OHdG levels in maternal blood, vital organs, placenta, and fetal tissues are shown in Figure 7 and Figure 8.

#### 3.7.1. Maternal Blood

At a 7.71 mg/kg dose, 8-OHdG peaked at 116.8 ± 4.3 ng/mL after 6 h, followed by 93.0 ± 5.2 ng/mL at 1 h and 84.5 ± 0.5 ng/mL at 24 h, compared with the control (16.8 ± 0.7 ng/mL) (Figure 7a). At 3.09 mg/kg, the highest level was 109.0 ± 2.7 ng/mL at 6 h, with 88.1 ± 8.7 ng/mL at 1 h and 79.2 ± 6.4 ng/mL at 24 h.

#### 3.7.2. Liver

At 7.71 mg/kg, liver homogenates showed maximal 8-OHdG (55.4 ± 15.9 ng/g) at 12 h, followed by 52.4 ± 14.3 ng/g at 6 h. Lower levels (~11.6 ng/g) were observed at 1 and 24 h, compared with the control (4.4 ± 0.1 ng/g) (Figure 7b). At 3.09 mg/kg, values ranged from 23.3 ng/g (1 h) to 13.5 ng/g (12 h).

#### 3.7.3. Spleen

At 3.09 mg/kg, splenic 8-OHdG levels were 31.3 ng/g (1 h), declining to ~11–14 ng/g at later time-points (Figure 7c). At 7.71 mg/kg, values ranged from 12.1 to 15.1 ng/g, compared with the control (7.7 ng/g).

#### 3.7.4. Placenta

At 3.09 mg/kg, placental 8-OHdG remained stable (12.4–13.7 ng/g) across all time-points, compared with the control (6.6 ng/g) (Figure 7d). At 7.71 mg/kg, the highest level (15.2 ± 0.2 ng/g) was observed at 24 h, with no significant differences at earlier intervals (13.1–13.8 ng/g).

#### 3.7.5. Fetus

At 7.71 mg/kg, fetal 8-OHdG peaked at 17.7 ± 0.9 ng/g at 12 h, followed by 14.7 ng/g (6 h), 13.2 ng/g (1 h), and 13.0 ng/g (24 h), compared with the control (7.7 ng/g) (Figure 7e). At 3.09 mg/kg, the highest level was 19.3 ± 0.2 ng/g at 1 h, with moderate values at 12 h (14.7 ng/g) and ~12 ng/g at 6 and 24 h.

#### 3.7.6. Correlation Analysis

Mean values of 8-OHdG levels across organs at the highest dose, 7.71 mg/kg, on the 19th day of gestation are illustrated in Figure 8a. Correlation analysis (Figure 8b) revealed a non-significant positive association between Zn^2+^ concentration in the same organs and 8-OHdG formation overall (R^2^ = 0.253). Liver showed no significant correlation (R^2^ = 0.018), while spleen exhibited a significant positive correlation (R^2^ = 0.611). Placenta (R^2^ = 0.162) and fetus (R^2^ = 0.301) showed non-significant associations.

## 4. Discussion

In this study, ZnONPs were synthesized following established protocols and characterized by >96% purity, distinct morphology, and strong crystallinity, consistent with previous reports [17]. Zeta potential analysis confirmed reduction in aggregation [18], while FTIR spectra matched earlier findings [19]. The stabilization and maintenance of the nanoscale size of ZnO nanoparticles (ZnONPs) in the present study can be explained by a combination of nucleation kinetics, surface charge effects, and surface passivation mechanisms.

First, ZnONP formation follows a classical nucleation–growth process, in which rapid nucleation relative to growth leads to the formation of many small nuclei, thereby limiting particle enlargement and favoring nanoscale dimensions. This behavior is consistent with the LaMer model, in which a burst of nucleation is followed by diffusion-limited growth, thereby restricting excessive increases in particle size [20].

Second, colloidal stabilization is largely governed by electrostatic repulsion. The measured zeta potential (−8.05 mV) indicates the presence of negatively charged surface groups, likely associated with adsorbed hydroxyl ions on the ZnO surface. These surface charges create an electrical double layer that generates interparticle repulsion, reducing aggregation in accordance with DLVO theory [21]. The FTIR band observed at 3398 cm^−1^ further supports the presence of surface hydroxyl groups contributing to this effect.

Third, surface hydroxylation and residual ionic species can act as weak capping or passivating agents, providing steric and chemical stabilization that limits particle growth and agglomeration. Such surface passivation mechanisms have been widely reported for ZnO nanostructures synthesized in aqueous systems [22].

Finally, the nanoscale nature of the particles is corroborated by XRD peak broadening, indicating small crystallite size, and TEM observations showing relatively uniform spherical nanoparticles (21.6–46.1 nm). These findings confirm that crystal growth was effectively constrained during synthesis. Overall, the nanoscale stability of ZnONPs in this study arises from controlled nucleation kinetics, electrostatic repulsion due to surface charge, and partial surface passivation by hydroxyl groups, all of which collectively prevent excessive growth and aggregation. Although the measured zeta potential (−8 mV) suggests limited electrostatic stabilization, the use of citrate buffer and supporting characterization analyses indicate that the ZnONPs maintained an acceptable dispersion state, while acknowledging the possibility of partial aggregation.

Toxicological evaluation classified ZnONPs as highly toxic following intravenous administration in female rats. During the 14-day acute toxicity trial, only minor cholinergic signs were observed, with no dose-dependent progression. Sub-lethal doses produce no overt toxicological effects. ZnONPs demonstrated rapid systemic distribution, penetrating maternal tissues, and crossing the placenta. Zn^2+^ was detected in all examined organs and fluids, with placental tissues showing the highest accumulation.

This transplacental transfer aligns with previous studies reporting fetal uptake of AgNPs, SiO_2_, and TiO_2_ nanoparticles after intravenous exposure [23,24]. Such findings reinforce concerns about nanoparticle transport across the placenta and its potential risks to fetal development and neonatal health [25].

Rat placental models are widely used to evaluate the developmental toxicity of drugs and chemicals, as rats share many similarities with humans in early placental development. However, notable differences exist, including variations in embryo-to-fetal period ratio, implantation type, yolk sac function, placental anatomy, and endocrine activity. Therefore, extrapolation of rat data to humans must be done cautiously, with careful consideration of these distinctions [26].

Maternal organs such as the liver, spleen, and kidney exhibited higher accumulation of ZnONPs than serum. This selective retention is likely due to zinc’s strong affinity for cysteine-rich proteins, particularly metallothioneins (MTs). MTs are low-molecular-weight proteins that chelate metal ions via sulfhydryl groups, thereby contributing to detoxification [27]. Their abundance in the kidney and spleen explains the preferential accumulation of ZnONPs in these tissues rather than in adipose or other organs. These findings are consistent with Abdel-Halim et al. [28], who reported significant accumulation of AgNPs in rat kidneys, and Hödl et al. [29], who noted high MT induction in Zn- and metal-exposed tissues. Overall, the biodistribution of ZnONPs is influenced by particle size, shape, concentration, and the duration of cellular contact [30].

The placenta displayed early high levels of ZnONPs at 1 h, followed by a sharp decline at 12 h (Table 1 and Table 2). However, the levels of ZnONPs in the placenta at 12 h and 24 h for the 7.71 mg/kg dose are 10–20 times lower compared to the 3.09 mg/kg dose. The reduced placental accumulation of ZnONPs observed at the higher dose (7.71 mg/kg) at 12 and 24 h may reflect dose-dependent alterations in nanoparticle biodistribution. At elevated concentrations, ZnONPs may undergo enhanced aggregation and increased sequestration by reticuloendothelial organs such as the liver and spleen, thereby reducing nanoparticle availability for placental uptake. In addition, saturation of placental transport mechanisms and accelerated systemic clearance may contribute to the reduced placental levels observed at later time points [24,31,32].

The lower zinc concentrations observed at the higher ZnONP dose, although seemingly counterintuitive, can be explained by dose-dependent biological and physicochemical processes. At higher nanoparticle concentrations, aggregation is more likely to occur, which reduces effective dispersion and bioavailability, thereby limiting tissue uptake. In addition, aggregated nanoparticles are more readily cleared by the reticuloendothelial system (RES), particularly in the liver and spleen, resulting in reduced measurable Zn levels in circulation and some tissues. Furthermore, nanoparticle toxicokinetics are often non-linear, and higher doses may trigger enhanced sequestration and clearance mechanisms that prevent proportional increases in tissue accumulation. Such non-linear dose–response behavior and enhanced clearance at higher nanoparticle doses have been previously reported in the literature [33].

Transplacental passage of ZnONPs has been associated with reduced fetal weight [11] and neurotoxicity [34]. The placenta plays an active, selective role in drug and chemical transport, with transfer influenced by membrane thickness, pore size, molecular weight, maternal blood pressure, and placental perfusion [35]. Diffusion is further determined by molecular size, ionic dissociation, and solubility [36]. Importantly, the novelty of the present work extends beyond the demonstration of placental transfer. This study provides a comprehensive, integrative evaluation of ZnONP toxicity in the maternal–placental–fetal axis, combining biodistribution, histopathology, and oxidative DNA damage assessment via 8-hydroxy-2′-deoxyguanosine (8-OHdG). To the best of our knowledge, the combined assessment of biodistribution, tissue injury, and oxidative DNA damage (8-OHdG) in a single in vivo maternal–fetal model has not been comprehensively reported for ZnONPs.

Several studies confirm the passage of nanoparticles from maternal blood to fetal tissues. For example, AuNPs sized 1.4–18 nm crossed the placenta after i.v. administration [37], while AgNPs were detected in fetuses following oral and i.v. exposure at gestation day 19 [23,28]. Particle size strongly influences transfer: large SiO_2_NPs (300–1000 nm) were absent in placenta and fetus [24], whereas smaller CdSe quantum dots (1.7 nm) showed higher tissue accumulation compared to larger counterparts [38].

Transplacental nanoparticle passage can result in adverse outcomes, including fetal growth restriction, malformations, and teratogenic effects. Independent of size, NPs can localize within mitochondria, nuclei, and cytoplasm, disrupting cellular functions [39]. ZnONP toxicity is mediated both indirectly, via Zn^2+^ ion release, and directly, through interactions between particles and tissues and organelles [40].

Histopathological analysis revealed adverse effects of ZnONPs on placental structure. Consistent with earlier reports, ZnONPs were shown to impair placental function, strongly associated with fetal growth restriction and miscarriage [41]. It was similarly reported that oral ZnONP exposure damaged placental architecture, reduced the spongiotrophoblast layer, and impaired function, leading to restricted fetal growth and reduced litter size.

Additional evidence from Abass et al. [42] demonstrated ZnONP-induced toxicity in albino rat thymus and spleen. Their findings included thickened splenic capsules and trabeculae, reduced white pulp compartments, and expanded red pulp due to congestion. Histological changes also included congested vessels, hemosiderin-laden cells, vacuolated splenocytes, darkly stained nuclei, and an increase in megakaryocytes. Splenic erythrophagocytosis and hemosiderosis may be exacerbated by persistent congestion, hemorrhage, hemolytic anemia, methemoglobinemia, or neoplastic conditions [43]. The association between ZnONPs and these pathological changes warrants further investigation.

The placenta barrier, nutritional transport, metabolic, and endocrine functions make it highly vulnerable to drug- or chemical-induced toxicity, and numerous placenta-toxic compounds have been identified [27]. In mammalian development, the placenta forms the maternal–fetal unit, and its dysfunction can result in abnormal fetal growth, resorption, or teratogenicity [44].

In this study, histological and ultrastructural examinations revealed ZnONP-induced cytotoxicity in maternal, placental, and fetal tissues. Trophoblastic giant cells at the maternal–placental interface, key endocrine regulators, were affected, consistent with their role in secreting prolactin-related hormones and cytokines [45]. Glycogen cells, distinct from spongiotrophoblasts, were also implicated; these transient basal zone cells contribute to glycogen metabolism and trophoblast invasion during mid-gestation [46]. Decidual zones undergo dynamic remodeling during pregnancy, with the decidua basalis forming a vascularized interface critical for maternal angiogenesis and immune modulation [47]. Disruption of these processes by ZnONPs parallels findings with AgNPs, where transplacental transfer induced genotoxicity in pregnant rats [16].

The identification of black inclusions as ZnO nanoparticles was based here on ultrastructural characteristics in addition to their treatment-dependent presence. At the ultrastructural level, these inclusions appeared as electron-dense (highly dark) particulate structures with nanoscale dimensions, which is a typical feature of metal-based nanoparticles due to their high electron-scattering properties. Their size range, morphology, and intracellular localization (e.g., within vesicular compartments consistent with endocytosis) agree with previously reported transmission electron microscopy (TEM) observations of ZnO nanoparticles in biological tissues [32]. Furthermore, these electron-dense inclusions were consistently observed only in treated samples and were absent in controls, supporting their association with the administered ZnONPs rather than endogenous structures. Nevertheless, techniques such as energy-dispersive X-ray spectroscopy (EDS), electron diffraction, or elemental mapping are required to conclusively confirm the presence and composition of ZnO nanoparticles.

Mechanistically, ZnONP toxicity is strongly linked to oxidative stress. Ultrafine particles generate reactive oxygen species (ROS), release Zn^2+^ ions, and physically interact with biomolecules. Excess ROS damages lipids, proteins, and nucleic acids, with hydroxyl radicals particularly reactive toward DNA, causing strand breaks, base oxidation, and adduct formation that may lead to mutagenesis and carcinogenesis [48].

Several published studies have demonstrated the in vivo toxicity of zinc oxide nanoparticles (ZnONPs) in animal models. Experimental evidence indicates that ZnONPs can induce oxidative stress, inflammation, and histopathological alterations in multiple organs, including the liver, brain, heart, and reproductive tissues. For instance, recent studies in rodents have shown that ZnONP exposure leads to significant oxidative damage and tissue injury in the hippocampus, heart, and testes [49], while other investigations reported mitochondrial dysfunction and neurotoxicity associated with increased reactive oxygen species generation [50]. Additionally in vivo studies have demonstrated hepatic and renal toxicity, disruption of zinc homeostasis, and DNA damage following ZnONP exposure. These findings collectively confirm that ZnONPs can exert dose-dependent and organ-specific toxic effects in vivo, largely mediated by oxidative stress and Zn^2+^ ion release [51].

8-Oxo-2’-deoxyguanosine (8-OHdG) is one of the most common ROS-induced DNA lesions in both nuclear and mitochondrial genomes and is widely recognized as a biomarker of oxidative stress. Numerous nanomaterials have been shown to induce toxicity through ROS generation [52]. In this study, ZnONP exposure significantly elevated 8-OHdG levels in maternal and fetal tissues, consistent with findings by Xiaoli et al. [34], who reported increased 8-OHdG in offspring brains following high-dose ZnONP exposure.

Elevated 8-OHdG is strongly associated with mutagenesis, carcinogenesis, and degenerative diseases due to its ability to induce GC→TA transversion mutations [52]. Beyond genotoxicity, ZnONPs demonstrated fetal toxicity, including reproductive and developmental impairment. Evidence suggests involvement of the Wnt/β-catenin pathway, with ZnONPs crossing the placental barrier, accumulating in the fetal liver, and inducing malformations [53]. Maternal exposure also reduced offspring brain weight, body weight, and survival index. Documented mechanisms include persistent neurotoxicity driven by oxidative stress, neuroinflammation, apoptosis, and epigenetic dysregulation, with histological confirmation of duration-dependent neuronal damage [54]. It is noteworthy to mention that the relatively low coefficients of determination (R^2^) observed in Figure 8 indicate relatively weak linear relationships between Zn^2+^ concentrations and 8-OHdG levels in some examined tissues. However, this finding is consistent with the multifactorial nature of oxidative DNA damage in biological systems. Specifically, 8-OHdG formation is not solely dependent on Zn^2+^ accumulation but is influenced by multiple interacting factors, including tissue-specific antioxidant capacity, metabolic activity, placental transfer dynamics, and local redox status. Therefore, a simple linear correlation is not expected to fully capture these complex biological interactions. Thus, the present associations could be interpreted as exploratory rather than causal. Further studies are warranted.

## 5. Conclusions

This study demonstrates that ZnO nanoparticles accumulate in maternal organs and are capable of crossing the placental barrier, leading to measurable fetal exposure. This distribution was associated with increased oxidative DNA damage, as indicated by elevated 8-OHdG levels in specific tissues. Notably, the observed effects exhibited tissue-dependent variability, suggesting differential susceptibility to ZnONP-induced oxidative stress. These findings provide experimental evidence of the biodistribution and biological impact of ZnONPs during pregnancy. While the results highlight potential toxicological concerns, particularly regarding maternal–fetal transfer, further studies are required to define dose–response relationships and long-term outcomes before extrapolating to human risk assessment.

## Figures and Tables

**Figure 1:**
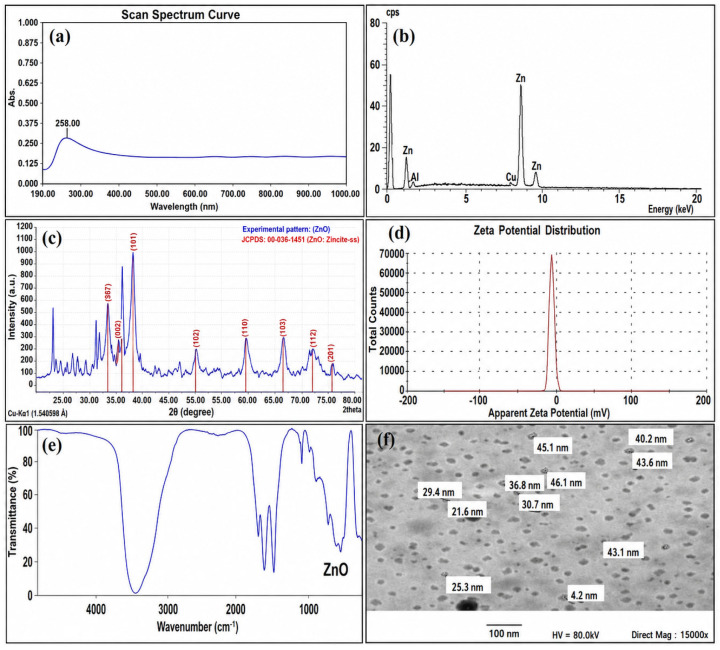
Characterization profiles of ZnONPs illustrate the following: (**a**) UV–Vis’s absorption spectrum of ZnONPs measured against a Milli-Q^®^ deionized water blank. Particles’ absorbance range was between 190 and 1000 nm; signals beyond this range were not captured. A baseline offset correction was applied to eliminate negative absorbance values arising from instrumental drift. (**b**) EDX analysis of ZnONPs, (**c**) XRD pattern of ZnONPs, (**d**) zeta potential analysis of the synthesized ZnONPs, (**e**) FTIR Spectrum of synthesized ZnONPs, and (**f**) an electron micrograph showing the ultrastructure of ZnONPs by TEM suspended in 1.0% citrate showing the majority of almost spherical-shaped NPs with diameters in the range 21.6–46.1 nm at 15,000×.

**Figure 2:**
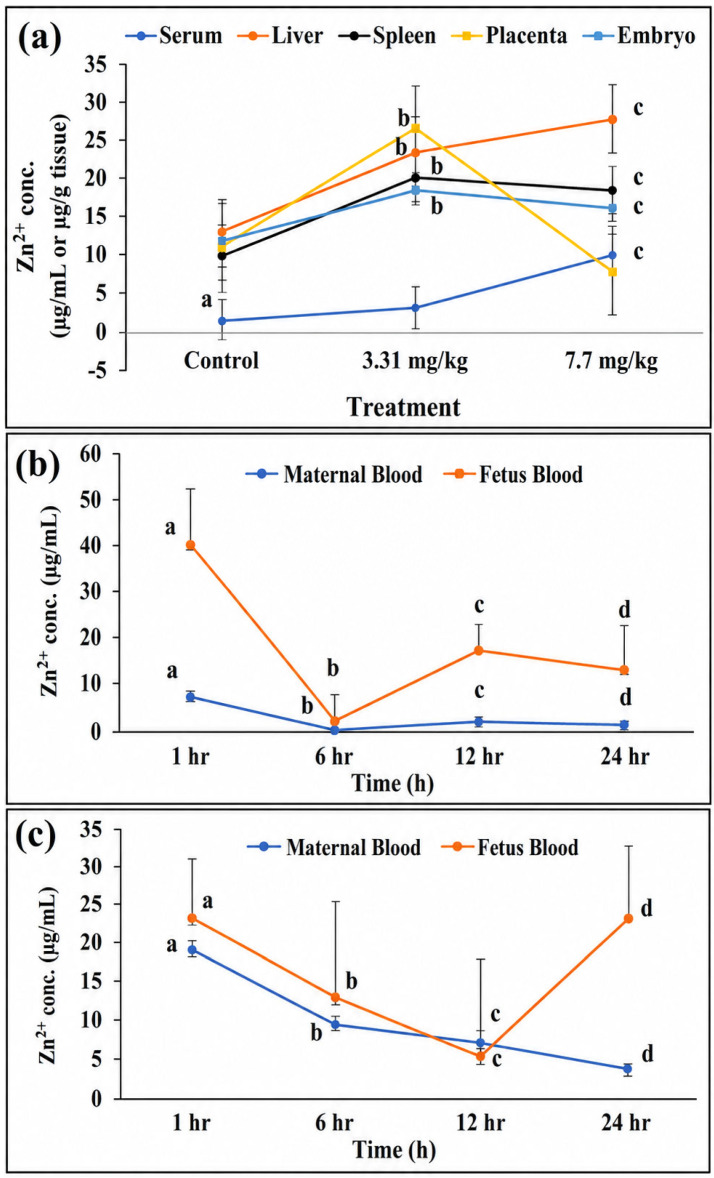
(**a**) Mean values of Zn^2+^ accumulation in selected organs following intravenous administration of ZnONPs at doses of 3.09 and 7.71 mg/kg in pregnant rats on gestational day 19. Zn^2+^ levels in maternal blood and fetal samples after intravenous administration of (**b**) 3.09 mg/kg and (**c**) 7.71 mg/kg ZnONPs at different time intervals. Data are presented as mean ± standard error (the mean value is from 5 separate measurements). Notably, greater variability was observed in fetal measurements compared to maternal blood, likely reflecting biological heterogeneity in placental transfer and fetal exposure. Values with different letters such as a, b, c, and d are significantly different at *p* < 0.05, while identical letters, such as a, a or b, b, etc., indicate no significant difference.

**Figure 3:**
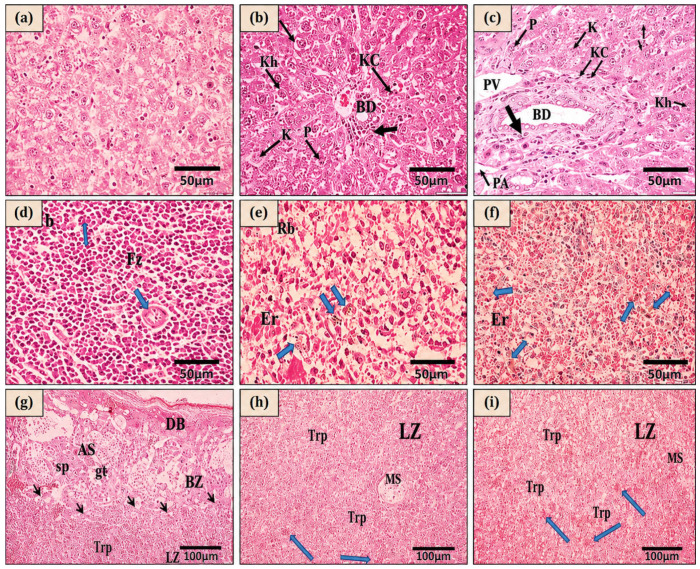
(**a**–**c**): Photomicrographs illustrate (**a**) normal hepatocytes of pregnant rats intravenously administered vehicle on the 19th day of gestation, (**b**) liver administered with 3.09 mg/kg of ZnONPs and sacrificed after 12 h, and (**c**) hepato-section of rats administered with 7.71 mg/kg of ZnONPs and sacrificed after 24 h. BD: bile ductule, KC: Kupffer cells, V: pale vacuolated, P: abnormal hepatocytes with pyknosis, Kh: karyorrhexis, K=: karyolysis in the nuclei of necrotic hepatocytes, PA: portal artery, PV: portal vein. H&E, ×400. (**d**–**f**): Photomicrographs illustrate (**d**) control splenic section, (**e**) splenic section from a rat administered with 3.09 mg/kg of ZnONPs and sacrificed after 12 h, and (**f**) splenic section from a rat administered with 7.71 mg/kg of ZnONPs and sacrificed after 24 h. H&E ×400. RP = red pulp, Er: erythrocytes, M: macrophages, and F: fibrotic area. (**g**–**i**): Photomicrographs illustrate (**g**) a section of placenta from a control pregnant rat, (**h**) a section of labyrinth zone (LZ) portion of placenta administered with 3.09 mg/kg of ZnONPs and sacrificed after 12 h, and (**i**) a section of a placenta administered with 7.71 mg/kg of ZnONPs and sacrificed after 12 h H&E. ×400. LZ: labyrinth zone, Trp: trophoblast cells, MS: maternal sinusoids, BZ: basal zone, DB: decidua basalis, sp: spongiotrophoblasts, gly: glycogen cells, FC: fetal capillary.

**Figure 4:**
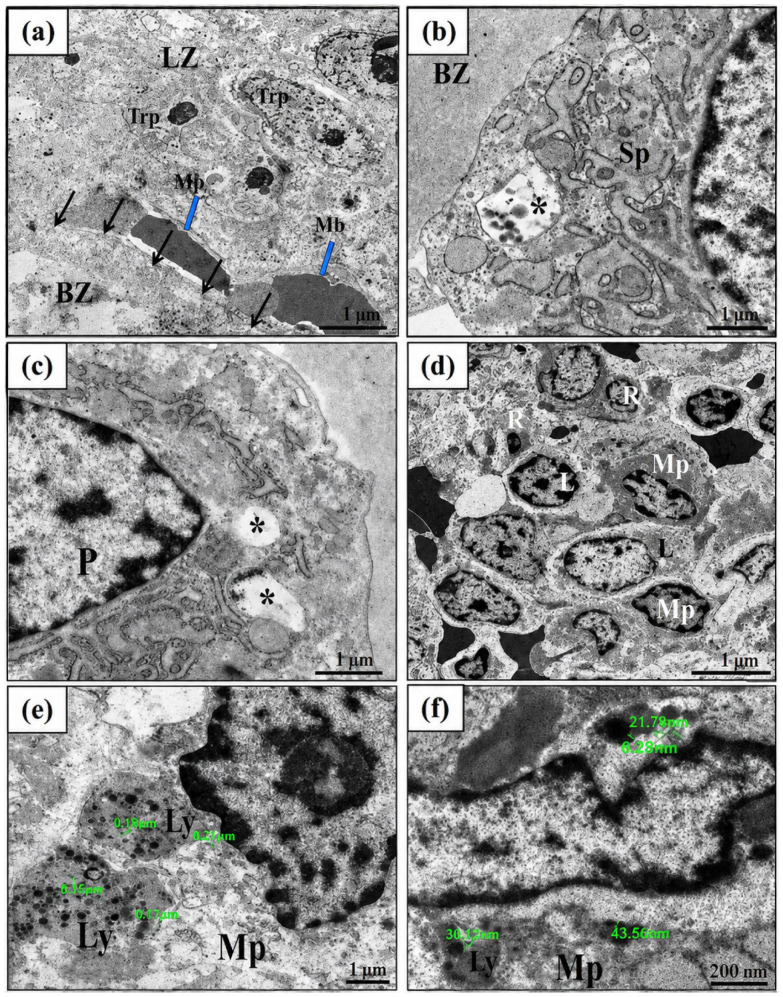
(**a**–**f**): Electron micrographs illustrate pathological changes or localization profiles of putative nanoparticles in the placenta and spleen. (**a**) Control placenta with normal structure, (**b**) placenta from a rat i.v administered with 3.09 mg/kg of ZnONPs and sacrificed after 12 h showing necrotic area (asterisk) containing presumed ZnONP deposits with sizes in a spongiotrophoblasts cell, and (**c**) shows trophoblasts (Trp) from a placenta of a pregnant rat treated with 7.71 mg/kg of ZnONPs and sacrificed after 12 h with necrotic properties such as chromatin condensation, some necrotic areas in cytoplasm (asterisk), and a pyknotic nucleus (P) [F_4_G_1_-OsO_4_ fixed-uranyl acetate lead citrate stained preparation, 8000×]. LZ = labyrinth zone, Trp = trophoblasts, Mb = maternal blood, BZ = basal zone, RER = rough endoplasmic reticulum, and V = vacuoles. (**d**–**f**): Electron micrographs illustrate (**d**) a section of normal spleen, (**e**) an electron micrograph of a spleen section from a rat i.v. administered with 3.09 mg/kg of ZnONPs and sacrificed after 12 h, showing localized putative nanoparticles, and (**f**) an electron micrograph of a spleen section from a rat treated with 7.71 mg/kg of ZnONPs and sacrificed after 12 h, with presumed localized nanoparticles [F_4_G_1_-OsO_4_ fixed-uranyl acetate lead citrate-stained preparation, 8000×]. L = numerous lymphocytes, Mp = macrophage, R = red pulp with reticular cells, V = vacuoles, ER = endoplasmic reticulum cristae.

**Figure 5:**
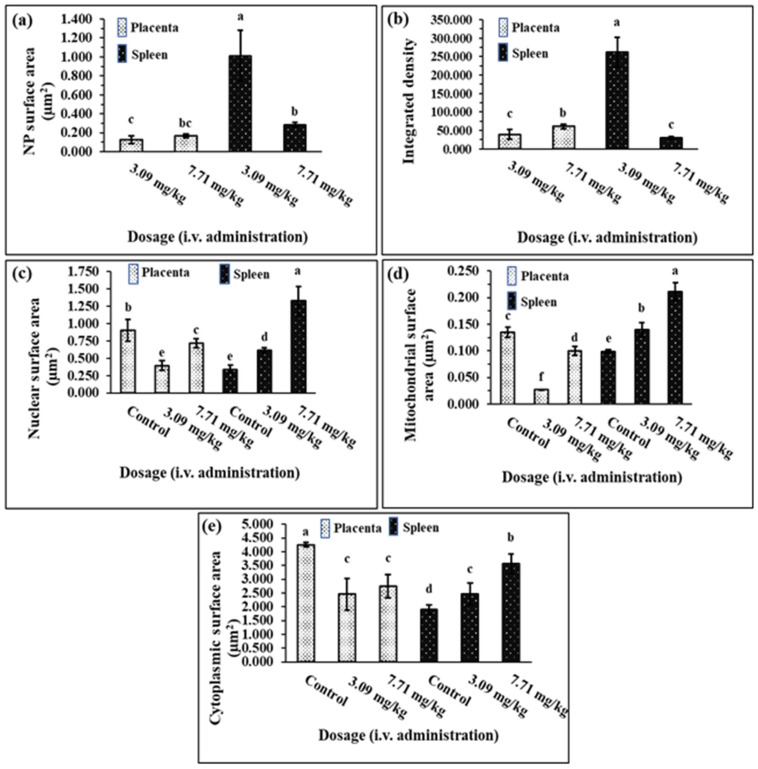
Quantitative morphometry of placenta and spleen of pregnant rats for (**a**) surface area (µm^2^) of engulfed NPs in lysosomes, (**b**) integrated density of precipitated ZnONPs following intravenous (i.v.) administration of 3.09 and 7.71 mg/kg body weight at the 19th day of gestation. Surface area (µm^2^) of (**c**) nuclei, (**d**) mitochondria, and (**e**) cytoplasm following i.v. doses. The mean value is from 5 separate measurements ± SD. Values with different letters such as a, b, c, and d are significantly different at *p* < 0.05, while identical letters, such as a, a; b, b; and c, c, etc., indicate no significant difference.

**Figure 6:**
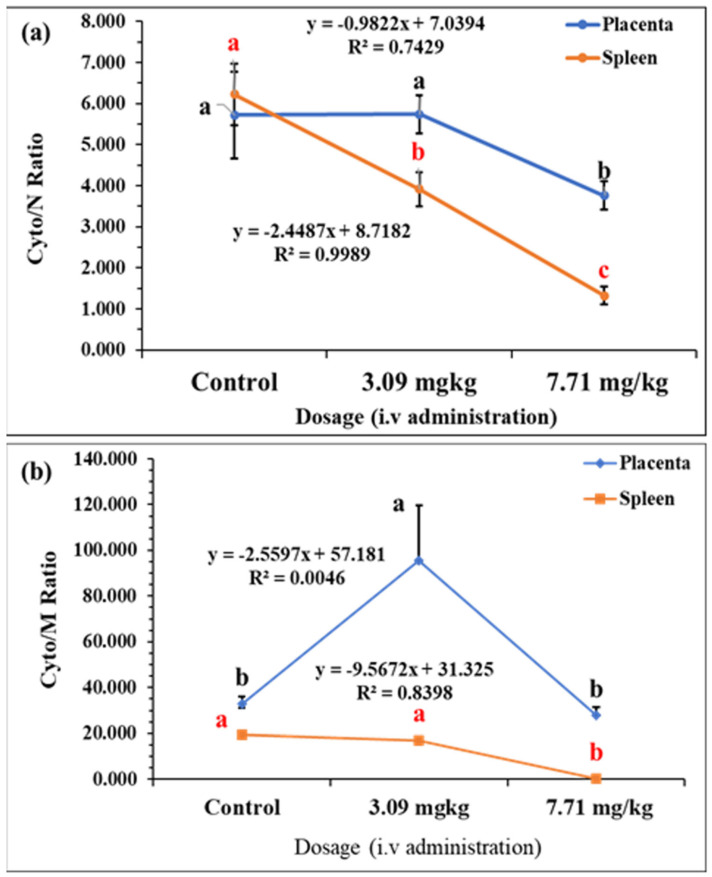
Quantitative morphometry calculations in the placenta and spleen of pregnant rats for (**a**) Cyto/N ratio with fitted regression line and (**b**) Cyto/M ratio following intravenous (i.v) doses: 3.09 and 7.71 mg/kg b. w of ZnONPs at the 19th day of gestation in a fitted regression line, and 95% prediction limits, and 95% confidence limits. The mean value is from 5 separate measurements± SD. Values with different letters, such as a, b, and c, are significantly different at *p* < 0.05, while identical letters, such as a, a or b, b, etc., indicate no significant difference.

**Figure 7:**
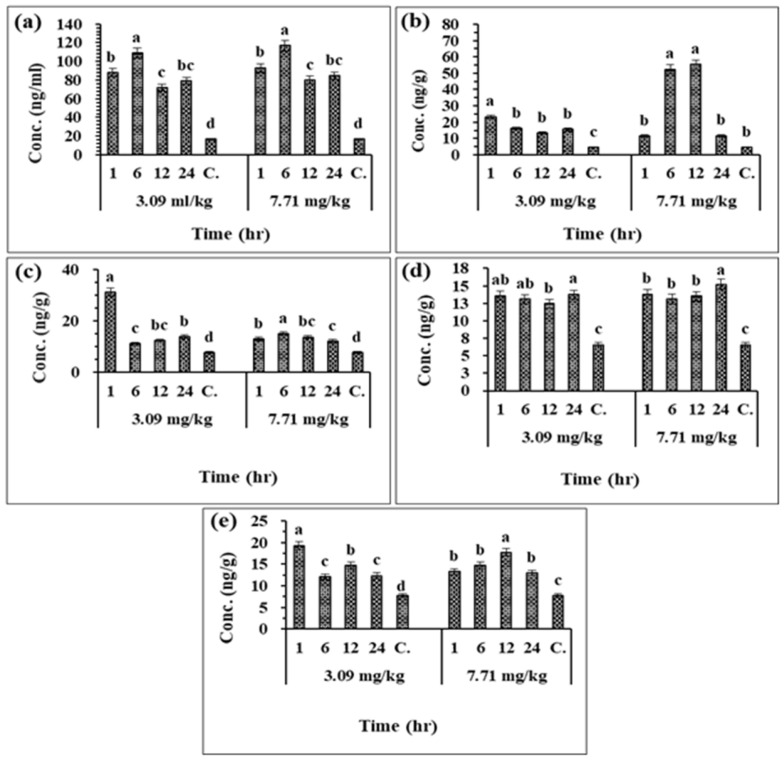
Levels of 8-OHdG at different time intervals in (**a**) serum, (**b**) liver, (**c**) spleen, (**d**) fetus, and (**e**) placenta of pregnant rats following intravenous (i.v) administration of ZnONPs (3.09 and 7.71 mg/kg b w) on the 19th day of gestation. The mean value is from 5 separate measurements± SD. Values with different letters, such as a, b, c, and d, are significantly different at *p* < 0.05, while identical letters, such as a, a; b, b, or bc, bc, indicate no significant difference.

**Figure 8:**
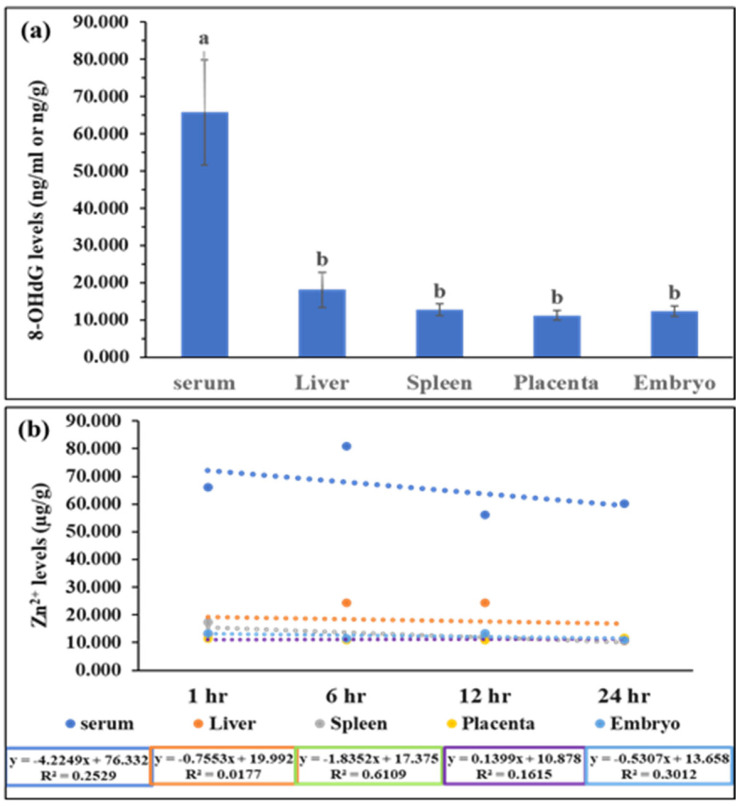
(**a**): Mean values of 8-OHdG levels (ng/mL or g) in the selected organs of pregnant rats following ZnONP administration on the 19th day of gestation. The mean value is from 5 separate measurements ± SD. Values with different letters such as a and b are significantly different at *p* < 0.05, while identical letters, such as b, b, indicate no significant difference. (**b**): Linear regression of ZnONP’ levels in the selected tissues and their 8-OHdG level in pregnant rats following intravenous administration of 7.71 mg/kg on the 19th day of gestation.

**Table 1:** Accumulation levels of ZnONPs (µg/mL or µg/g) in selected tissues after 3.09 mg/kg bw intravenous (i.v.) administration in pregnant rats for time-point intervals.

Time (h)	Serum (µg/mL)	Liver (µg/g)	Kidney (µg/g)	Spleen (µg/g)	Placenta (µg/g)	Uterus (µg/g)	Amniotic Fluid (µg/mL)	Embryo (µg/g)
**1**	7.80 ± 0.80 **^bc^**	13.40 ± 2.40 **^b^**	49.40 ± 9.10 **^a^**	50.70 ± 4.50 **^a^**	29.00 ± 5.90 **^ab^**	46.50 ± 3.60 **^ab^**	0.30 ± 0.10 **^e^**	40.70 ± 12.3 **^a^**
**6**	0.50 ± 0.02 **^e^**	25.60 ± 0.70 **^ab^**	13.90 ± 0.20 **^b^**	8.00 ± 0.50 **^b^**	21.20 ± 1.10 **^abc^**	65.20 ± 0.50 **^a^**	0.70 ± 0.10 **^de^**	3.02 ± 5.10 **^b^**
**12**	2.30 ± 0.20 **^de^**	40.70 ± 0.30 **^b^**	10.40 ± 1.40 **^b^**	16.90 ± 5.40 **^b^**	10.50 ± 3.50 **^bc^**	3.97 ± 2.10 **^e^**	1.60 ± 0.00 **^de^**	17.40 ± 5.40 **^b^**
**24**	1.96 ± 0.70 **^e^**	15.20 ± 6.50 **^b^**	BDL	5.90 ± 2.10 **^b^**	45.60 ± 9.60 **^a^**	15.10 ± 5.00 **^de^**	43.60 ± 0.40 **^a^**	13.50 ± 8.70 **^b^**
**LSD_0.05_**	5.21	20.79	34.94	30.42	25.37	23.18	4.936	N.S

Each value represents the mean of six treated animals ± S.D.; BDL: Below detection level; and LSD: Least significant difference. Values with different superscript letters such as a, b, c, and d are significantly different at *p* < 0.05, while identical letters, such as a, a or b, b, etc., indicate no significant difference.

**Table 2:** Accumulation levels of ZnONPs (µg/mL or g) in selected tissues after 7.71 mg/kg bw intravenous (i.v.) administration in pregnant rats for time-point intervals.

Time (h)	Serum (µg/mL)	Liver (µg/g)	Kidney (µg/g)	Spleen (µg/g)	Placenta (µg/g)	Uterus (µg/g)	Amniotic Fluid (µg/mL)	Embryo (µg/g)
**1**	19.16 ± 1.3 **^a^**	9.2 ± 0.8 **^b^**	30.0 ± 9.7 **^ab^**	11.4 ± 0.5 **^b^**	9.7 ± 1.03 **^bc^**	41.7 ± 6.4 **^bc^**	9.9 ± 1.5 **^bc^**	23.2 ± 8.1 ^a^
**6**	9.9 ± 1.03 **^b^**	19.6 ± 0.6 **^b^**	24.1 ± 2.9 **^ab^**	BDL	20.3 ± 3.4 **^bc^**	22.5 ± 4.2 **^cde^**	4.4 ± 0.2 **^de^**	13.1 ± 12.4 ^b^
**12**	7.4 ± 1.6 **^bcd^**	44.3 ± 3.4 **^a^**	17.4 ± 2.3 **^ab^**	33.7 ± 9.6 **^ab^**	0.1 ± 0.8 **^c^**	32.4 ± 4.4 **^bcd^**	5.5 ± 0.4 **^cd^**	5.7 ± 12.3 ^b^
**24**	4.1 ± 0.51 **^cde^**	38.3 ± 6.7 **^a^**	19.5 ± 3.0 **^ab^**	29.4 ± 8.9 **^ab^**	1.9 ± 1.7 **^c^**	21.1 ± 3.4 **^cde^**	13.1 ± 1.8 **^b^**	23.2 ±9.6 ^a^
**LSD**	5.21	20.79	34.94	30.42	25.37	23.18	4.936	N.S

Each value represents the mean of six treated animals ± S.D.; BDL: Below detection level; and LSD: Least significant difference. Values with different superscript letters, such as a, b, c, ab, bc, etc., are significantly different at *p* < 0.05, while identical letters, such as a, a; b, b; or ab, ab, etc., indicate no significant difference.

**Table 3:** Placental transfer % of ZnONPs in pregnant rats on 19th day of gestation following intravenous (i.v) administration of 3.09 and 7.71 mg/kg for time-point intervals.

Time-Point (hr)	Placental Transfer %		
	3.09 mg/kg	7.71 mg/kg	Time Mean %
1	6.14 ± 1.56 ^a^	1.6 ± 0.51 ^b^	3.87 ± 1.04
6	1.49 ± 0.48 ^b^	1.42 ± 0.65 ^b^	1.45 ± 0.39
12	3.07 ± 0.92 ^ab^	0.95 ± 0.6 ^b^	2.00 ± 0.61
24	3.08 ± 1.05 ^ab^	1.65 ± 0.54 ^b^	2.37 ± 0.60
LSD (0.05)	3.483		2.463
Treatment Mean	3.45 ± 0.61	1.4 ± 0.28	
LSD (0.05)	1.741		

LSD: Least significant difference. Values with different letters, such as a and b, are significantly different at *p* < 0.05, while identical letters, such as b, b, or ab, ab, indicate no significant difference.

## Data Availability

The raw data supporting the conclusions of this article will be made available by the authors on request.

## Abbreviations

8-OHdG: 8-Hydroxydeoxyguanosine, ANOVA: Analysis of Variance, CV: Coefficient of Variation, EDX: Energy-Dispersive X-ray Spectroscopy, ELISA: Enzyme-Linked Immunosorbent Assay, FTIR: Fourier-Transform Infrared Spectroscopy, H&E: Hematoxylin and Eosin (staining), i.v.: Intravenous, ICP-OES: Inductively Coupled Plasma Optical Emission Spectroscopy, LD_50_: Median Lethal Dose, LSD: Least Significant Difference, NMs: Nanomaterials, PBS: Phosphate-Buffered Saline, SEM: Scanning Electron Microscope, TEM: Transmission Electron Microscopy, XRD: X-ray Diffraction, ZnONPs: Zinc Oxide Nanoparticles.

## References

[1] Singh A., Kim H., Park J. (2026). Advances in metallic and metal oxide nanoparticles: Synthesis, properties, and applications in electronics and biomedicine. J. Nanosci. Nanotechnol..

[2] Raha S., Ahmaruzzaman M. (2022). ZnO nanostructured materials and their potential applications: Progress, challenges and perspectives. Nanoscale Adv..

[3] Saad A.M. (2023). Chemical extraction and quantification of extracellular polymeric substances in unspiked-metaldehyde and spiked-metaldehyde of rubber aerobic granular sludge and molasses aerobic granular sludge. Environ. Eng. Res..

[4] Yang H., Niu S., Guo M., Liu C., Xue Y. (2025). Toxic mechanisms of nanoparticle-induced ferroptosis and current research challenges: A critical review. Environ. Pollut..

[5] Wang Z., Wang Z. (2020). Nanoparticles induced embryo-fetal toxicity. Toxicol. Ind. Health.

[6] Hou J., Wu Y., Li X., Wei B., Li S., Wang X. (2018). Toxic effects of different types of zinc oxide nanoparticles on algae, plants, invertebrates, vertebrates and microorganisms. Chemosphere.

[7] Nagar V., Singh T., Tiwari Y., Aseri V., Pandit P., Chopade R., Pandey K., Lodha P., Awasthi G. (2022). ZnO Nanoparticles: Exposure, toxicity mechanism and assessment. Mater. Today Proc..

[8] Srivastava A.K., Yadav S.S., Mishra S., Yadav S.K., Parmar D., Yadav S. (2020). A combined microRNA and proteome profiling to investigate the effect of ZnO nanoparticles on neuronal cells. Nanotoxicology.

[9] Fujihara J., Nishimoto N. (2024). Review of Zinc Oxide Nanoparticles: Toxicokinetics, Tissue Distribution for Various Exposure Routes, Toxicological Effects, Toxicity Mechanism in Mammals, and an Approach for Toxicity Reduction. Biol. Trace Elem. Res..

[10] Campagnolo L., Lacconi V., Bernardini R., Viziano A., Pietroiusti A., Ippoliti L., Moleti A., Sisto R. (2024). Maternal exposure to zinc oxide nanoparticles causes cochlear dysfunction in the offspring. Front. Toxicol..

[11] Morales-Rubio R.A., Alvarado-Cruz I., Manzano-León N., Andrade-Oliva M.D., Uribe-Ramirez M., Quintanilla-Vega B., Osornio-Vargas Á., De Vizcaya-Ruiz A. (2019). In utero exposure to ultrafine particles promotes placental stress-induced programming of renin-angiotensin system-related elements in the offspring results in altered blood pressure in adult mice. Part. Fibre Toxicol..

[12] Pacholski C., Kornowski A., Weller H. (2002). Self-Assembly of ZnO: From Nanodots to Nanorods. Angew. Chem. Int. Ed. Engl..

[13] Schmickler W., Santos E. (2010). Interfacial Electrochemistry.

[14] OECD (1987). Test No. 402: Acute Dermal Toxicity.

[15] Finney D.J. (1971). Probit Analysis: A Statistical Treatment of the Sigmoid Response Curve.

[16] Salim E.I., Abdel-Halim K.Y., El-Mahalawy M.E., Badr H.A., Ahmed H. (2023). Tissue Distribution, Pharmacokinetics, and Effect of Hematological and Biochemical Parameters of Acute Intravenous Administration of Silver Nanoparticles in Rats. Nanomaterials.

[17] Albarakaty F.M., Alzaban M.I., Alharbi N.K., Bagrwan F.S., Abd El-Aziz A.R.M., Mahmoud M.A. (2023). Zinc oxide Nanoparticles, Biosynthesis, characterization and their potentphotocatalytic degradation, and antioxidant activities. J. King Saud. Univ. Sci..

[18] Al-Kordy H.M.H., Sabry S.A., Mabrouk M.E.M. (2021). Statistical optimization of experimental parameters for extracellular synthesis of zinc oxide nanoparticles by a novel haloalaliphilic *Alkalibacillus* sp. W7. Sci. Rep..

[19] Yedurkar S., Maurya C., Mahanwar P. (2016). Biosynthesis of Zinc Oxide Nanoparticles Using Ixora Coccinea Leaf Extract—A Green Approach. Open J. Synth. Theory Appl..

[20] Sugimoto T. (2000). Fine Particles: Synthesis, Characterization, and Mechanisms of Growth.

[21] Bhattacharjee S. (2016). DLS and zeta potential—What they are and what they are not?. J. Control. Release.

[22] Baruah S., Dutta J. (2009). Hydrothermal Growth of ZnO Nanostructures. Sci. Technol. Adv. Mater..

[23] Salim E.I., Abdel-Halim K.Y., Abu-Risha S.E., Abdel-Latif A.S. (2019). Induction of 8-hydroxydeoxyguanosine and ultrastructure alterations by silver nanoparticles attributing to placental transfer in pregnant rats and fetuses. Hum. Exp. Toxicol..

[24] Yamashita K., Yoshikoka Y., Higashisaka K., Mimura K., Morishita Y., Nozaki M., Yoshida T., Ogura T., Nabeshi H., Nagano K. (2011). Silica and titanium dioxide nanoparticles cause pregnancy complications in mice. Nat. Nanotechnol..

[25] Yokel R.A., MacPhail R.C. (2011). Engineered nanomaterials: Exposures, hazards, and risk prevention. J. Occup. Med. Toxicol..

[26] Furukawa S., Hayashi S., Usuda K., Abe M., Hagio S., Ogawa I. (2011). Toxicological Pathology in the Rat Placenta. J. Toxicol. Pathol..

[27] Liu Y., Wu Z., Guo K., Zhou Y., Xing K., Zheng J., Sun Y., Zhang J. (2022). Metallothionein-1 gene from Exopalaemon carinicauda and its response to heavy metal ions challenge. Mar. Pollut. Bull..

[28] Abdel-Halim K.Y., Salim E.I., Abdel-Latif A.S., Abu-Risha S.E. (2023). Tissue distribution, placental transfer and excretion of silver nanoparticles in pregnant rats after a single oral dose. Environ. Anal. Health Toxicol..

[29] Hödl E., Felder E., Chabicovsky M., Dallinger R. (2010). Cadmium stress stimulates tissue turnover in Helix pomatia: Increasing cell proliferation from metal tolerance to exhaustion in molluscan midgut gland. Cell Tissue Res..

[30] Dey S., Mohanty D.L., Divya N., Bakshi V., Mohanty A., Rath D., Das S., Mondal A., Roy S., Sabui R. (2025). A critical review on zinc oxide nanoparticles: Synthesis, properties and biomedical applications. Intell. Pharm..

[31] De Jong W.H., Hagens W.I., Krystek P. (2008). Particle size-dependent organ distribution of gold nanoparticles after intravenous administration. Biomaterials.

[32] Muoth C., Aengenheister L., Kucki M., Wick P., Buerki-Thurnherr T. (2016). Nanoparticle transport across the placental barrier: Pushing the field forward!. Nanomedicine.

[33] Chen Q., Riviere J.E., Lin Z. (2022). Toxicokinetics, dose-response, and risk assessment of nanomaterials: Methodology, challenges, and future perspectives. Wiley Interdiscip. Rev. Nanomed. Nanobiotechnology.

[34] Xiaoli F., Junrong W., Xuan L., Yanli Z., Limin W., Jia L., Longquan S. (2017). Prenatal exposure to nanosized zinc oxide in rats: Neurotoxicity and postnatal impaired learning and memory ability. Nanomedicine.

[35] Vaupel P., Multhoff G. (2022). Blood flow and respiratory gas exchange in the human placenta at term: A data update. Adv. Exp. Med. Biol..

[36] Cary C., Stapleton P. (2023). Determinants and mechanisms of inorganic nanoparticle translocation across mammalian biological barriers. Arch. Toxicol..

[37] Semmler-Behnke M., Fertsch S., Schmide O., Wenk A., Kreyling W. (2007). Uptake of 1.4 nm versus 18 nm gold nanoparticles in secondary target organs is size dependent in control and pregnant rats after intratracheal or intravenous application. Euro Nano Forum.

[38] Adhipandito C.F., Cheung S.H., Liu Y.H., Wu S.H. (2021). Atypical renal clearance of nanoparticles larger than the kidney filtration threshold. Int. J. Mol. Sci..

[39] Raoof M., Mackeyev Y., Cheney M.A., Wilson L.J., Curley S.A. (2012). Internalization of C60 fullerenes into cancer cells with accumulation in the nucleus via the nuclear pore complex. Biomaterials.

[40] Acharya B., Behera A., Moharana S., Prajapati B.G., Behera S. (2025). Nanoparticle-mediated embryotoxicity: Mechanisms of chemical toxicity and implications for biological development. Chem. Res. Toxicol..

[41] Chen B., Hong W., Yang P., Tang Y., Zhao Y., Aguilar Z.P., Xu H. (2020). Nano zinc oxide induced fetal mice growth restriction, based on oxide stress and endoplasmic reticulum stress. Nanomaterials.

[42] Abass M.A., Selim S.A., Selim A.O., El-Shal A.S., Gouda Z.A. (2017). Effect of orally administered zinc oxide nanoparticles on albino rat thymus and spleen. IUBMB Life.

[43] Abu-Gharbia M.A., El-Sayed M.F., Salem J.M., Abd-Elsamei W.M., Al-Arabi G. (2025). Antibacterial impact of biosynthesized zinc oxide nanoparticles on uropathogenic Escherichia coli and in vivo assessment of physiological and histological alterations. Sci. Rep..

[44] Yang H., Sun C., Fan Z., Tian X., Yan L., Du L., Liu Y., Chen C., Liang X.J., Anderson G.J. (2012). Effects of gestational age and surface modification on materno-fetal transfer of nanoparticles in murine pregnancy. Sci. Rep..

[45] Ladyman S.R., Georgescu T.R., Stewart A.M., Khant Aung Z., Cheong I., Grattan D.R., Brown R.S.E. (2025). Prolactin modulation of thermoregulatory circuits provides resilience to thermal challenge of pregnancy. Cell Rep..

[46] Rodrigues-Machado M.G., Silva G.C., Pinheiro M.B., Caliari M.V., Borges E.L. (2010). Effects of sepsis-induced acute lung injury on glycogen content in different tissues. Exp. Lung Res..

[47] Jones R.L., Stoikos C., Findlay J.K., Salamonsen L.A. (2006). TGF-beta superfamily expression and actions in the endometrium and placenta. Reproduction.

[48] Akter S., Madhuvilakku R., Kar A.K., Nila I.S., Liu P., Inuzuka H., Wei W., Hong Y. (2026). Reactive oxygen species (ROS) in cancer: From mechanism to therapeutic implications. Signal Transduct. Target. Ther..

[49] Abdelkhalek N.K.M., Ghazy E.W., Abdel-Daim M.M. (2019). Pharmacodynamic interaction of zinc oxide nanoparticles with various organs in vivo: Oxidative stress and histopathological changes. Environ. Sci. Pollut. Res. Int..

[50] Liu H., Yang D., Yang H., Zhang H., Zhang W., Fang Y., Lin Z., Tian L., Lin B., Yan J. (2013). Comparative study of respiratory tract immune toxicity induced by three sterilisation nanoparticles: Silver, zinc oxide and titanium dioxide. J. Hazard. Mater..

[51] Vandebriel R.J., De Jong W.H. (2012). A review of mammalian toxicity of ZnO nanoparticles. Nanotechnol. Sci. Appl..

[52] Omari S.S., Nasirzadeh N. (2021). 8-Hydroxy-2’-deoxyguanosine (8-OHdG) as a biomarker of oxidative DNA damage induced by occupational exposure to nanomaterials: A systematic review. Nanotoxicology.

[53] Qing X., Shi D., Lv X., Wang B., Chen S., Shao Z. (2019). Prognostic significance of 8-hydroxy-2′-deoxyguanosine in solid tumors: A meta-analysis. BMC Cancer.

[54] Wei X., Liu O., Wang Z., Xu G. (2024). Zinc oxide nitrogen oxide crosses the placental barrier and induces fetal malformations through activation of the Wnt/β-catenin signaling pathway. J. Nanopart. Res..

